# Comparative Genomics of the First Resistant *Candida auris* Strain Isolated in Mexico: Phylogenomic and Pan-Genomic Analysis and Mutations Associated with Antifungal Resistance

**DOI:** 10.3390/jof10060392

**Published:** 2024-05-30

**Authors:** Arturo Casimiro-Ramos, Celia Bautista-Crescencio, Alvaro Vidal-Montiel, Gloria M. González, Juan Alfredo Hernández-García, César Hernández-Rodríguez, Lourdes Villa-Tanaca

**Affiliations:** 1 Laboratorio de Biología Molecular de Bacterias y Levaduras, Departamento de Microbiología, Escuela Nacional de Ciencias Biológicas, Instituto Politécnico Nacional, Prolongación de Carpio y Plan de Ayala, Casco de Santo Tomás, Ciudad de México 11340, Mexico; acasimiror1800@alumno.ipn.mx (A.C.-R.); luna_20_08@hotmail.com (C.B.-C.); alvaro301013@gmail.com (A.V.-M.); freddyhardcore@gmail.com (J.A.H.-G.); chdez38@hotmail.com (C.H.-R.); 2 Departamento de Microbiología, Facultad de Medicina, Universidad Autónoma de Nuevo León, Hospital Universitario “Dr. José Eleuterio Gonzalez”, Av. Madero y Calle Dr. Eduardo Aguirre Pequeño s/n, Colonia Mitras Centro, Monterrey 64460, Nuevo Leon, Mexico; gloria.gonzalezgn@uanl.edu.mx

**Keywords:** *Candida auris*, whole genome sequencing (WGS), phylogenomics, pan-genome analysis, azole resistance, K143R, Erg 11 mutation

## Abstract

*Candida auris* is an emerging multidrug-resistant and opportunistic pathogenic yeast. Whole-genome sequencing analysis has defined five major clades, each from a distinct geographic region. The current study aimed to examine the genome of the *C. auris* 20–1498 strain, which is the first isolate of this fungus identified in Mexico. Based on whole-genome sequencing, the draft genome was found to contain 70 contigs. It had a total genome size of 12.86 Mbp, an N50 value of 1.6 Mbp, and an average guanine-cytosine (GC) content of 45.5%. Genome annotation revealed a total of 5432 genes encoding 5515 proteins. According to the genomic analysis, the *C. auris* 20–1498 strain belongs to clade IV (containing strains endemic to South America). Of the two genes (*ERG11* and *FKS1*) associated with drug resistance in *C. auris*, a mutation was detected in K143R, a gene located in a mutation hotspot of *ERG11* (lanosterol 14-α-demethylase), an antifungal drug target. The focus on whole-genome sequencing and the identification of mutations linked to the drug resistance of fungi could lead to the discovery of new therapeutic targets and new antifungal compounds.

## 1. Introduction

*Candida auris* was first reported in Japan in 2009 after being isolated from the secretion of the external ear canal of a female patient [1]. Subsequently, clinical isolates of the same strain in South Korea were retrospectively identified, dating back to 1996 [2]. In a little over a decade, *C. auris* has emerged in healthcare settings worldwide and is suggested to be involved in numerous medical conditions. It is capable of colonizing the skin and causing outbreaks of invasive candidiasis. Bloodstream infections are the most frequent invasive condition and have been responsible for increasing in-hospital mortality rates by up to 70% [3,4].

As part of the public health response, whole-genome sequencing (WGS) has played a significant role in characterizing the transmission dynamics of *C. auris* and in detecting new outbreaks [3]. With this technique, it has been possible to define five clades and one potential sixth clade of *C. auris*. Clade I is manifested in South Asia, II in East Asia, III in Africa, IV in South America, and V in Iran [4,5,6]. A possible clade VI has been proposed based on three *C. auris* isolates found in Singapore that are genetically distinct from clades I–V [7].

Each clade has a specific level of resistance to the main antifungals administered to treat candidiasis (azoles, echinocandins, and amphotericin B). Isolates belonging to clade II are typically susceptible to azoles. At the same time, resistance to these drugs is shown by nearly all the isolates in clades I and III and by roughly half of those in clade IV. Some of the resistant isolates contain one of three mutations (F126L, Y132F, and K143R) in lanosterol 14-⍺-demethylase (ERG11), a drug target [3,4,8,9].

A small percentage of the isolates from clades I, III, and IV are resistant to echinocandins. Resistance has been linked to a single mutation at S639 (S639Y/P/F) in the hotspot 1 region of 1,3-β-D-glucan synthase (Fks1), another drug target [3,9]. Although low susceptibility to amphotericin B is common in clades I and IV, the mutations responsible for drug-resistance have not been defined [8]. In Mexico, *C. auris* 20–1498 was first isolated from a blood sample of a patient with gastrointestinal complications and endometriosis in May of 2020. This strain was identified by sequence analysis of the ITS1-5.8S-ITS2 and D1/D2 ribosomal regions [10].

The current contribution aimed to determine the genome of the *C. auris* 20–1498 strain, confirm the clade to which it belongs by comparative genomics, and analyze the mutations responsible for its resistance to azoles. Information on the genome of this strain will allow for its comparison with the genome of the *C. auris* strain isolated months later in the same hospital and associated with COVID-19.

## 2. Materials and Methods

### 2.1. Clinical Data and Isolates

The *C. auris* 20–1498 strain was isolated from a blood culture of a 58-year-old woman with severe endometriosis (stage IV). The patient had a history of multiple hospitalizations and the application of a central catheter and Mahurkar catheter. The *C. auris* 20–1498 isolate was identified by the Microbiology Department of the Faculty of Medicine in the Universidad Autónoma de Nuevo Leon in conjunction with the University Hospital (Hospital Universitario Dr. Jose Eleuterio Gonzalez) [10].

### 2.2. Fungal Growth Conditions

The *C. auris* 20–1498 isolate was cultured on Sabouraud dextrose agar plates (SDA; MCD LAB, S.A. de C.V., Mexico) at 35 °C for 2 days. The morphology and purity of the *C. auris* 20–1498 colony were established on solid SDA.

### 2.3. DNA Extraction and Genome Sequencing

For the genomic identification of the *C. auris* 20–1498 isolate, DNA was extracted with the Zymo Research^®^ Soil Microbe DNA Miniprep kit, Irvine, CA, USA. Whole-genome sequencing was carried out on the Illumina HiSeq 4000 system (Novogene, Sacramento, CA, USA).

### 2.4. Thermotolerance and Halotolerance

The thermotolerance of *C. auris* 20–1498 growth was tested according to the modified protocol described by Reséndiz-Sánchez et al. in 2020, using *C. haemulonii* 87, *C. albicans* ATCC 10231, *C. glabrata* CBS 138, and *C. auris* CJ97 as controls. The yeasts were grown in yeast extract peptone dextrose (YPD) broth under constant shaking at 28 °C until reaching the early stationary growth phase (~15 h). The inoculum was adjusted to As_600_ = 0.5 with sterile YPD medium, and 5 μL of each strain was inoculated into the corresponding culture medium and streaked with a microbiological loop. The cultures were incubated at different temperatures (28, 37, and 42 °C), and yeast growth was recorded every 24 h for 3 days. The solid culture media utilized were SDA, YPD, YPD with 1 M NaCl, YPD with 2 M NaCl, and blood agar [11]. *C. haemulonii* was sensitive at temperatures of 37 and 42 °C in SDA medium. In contrast, *C. albicans* and *C. auris* could grow at all three tested temperatures. 

### 2.5. Genome Assembly and Annotation

The whole-genome sequencing reads were assessed for quality with the FastQC v0.11.9 program [12] and then trimmed with Trimmomatic v0.39 [13]. Subsequently, the genome was assembled on Velvet v1.2.10 software by means of the referenced assembly method, with the *C. auris* B11220 strain (GCA_003013715.2) as the reference genome [14]. Finally, the assembly quality was evaluated with the QUAST v5.0.2 program [15]. The genome annotation of *C. auris* 20–1498 was achieved on the Companion v1.0.2 server [16], using *C. auris* B8441 as the reference strain to standardize the models for gene finding, functional annotation transfer, and pseudochromosome contiguation. The *C. auris* 20–1498 genome sequence and gene annotation generated were deposited in the GenBank BioProject (access number: PRJNA1013603) and BioSample ID (SAMN39051800). 

### 2.6. Phylogenomic Tree and Pan-Genome of Candida auris

The phylogenomic tree contains a total of twenty-six *C. auris* yeast genomes. The genome sequences of the *C. auris* isolates were downloaded from the NCBI database [17]: B11205 (GCA_016772135.1), B8441 (GCA_002759435.2), B13916 (GCA_016772235.1), 20–26 (GCA_025429755.1), 20–32 (GCA_025429595.1), BJCA001 (GCA_018831645.1), CA8LBN (GCA_019039635.1), CA27LBN (GCA_019039335.1), L1537/2020 (GCA_020809265.1), RCPF-1821 (GCA_004287075.1), B12043 (GCA_016495645.1), B11809 (GCA_016495685.1), B13463 (GCA_016495665.1), B11220 (GCA_003013715.2), B11221 (GCA_002775015.1), B12037 (GCA_016772215.1), B12631 (GCA_016772195.1), BJCA002 (GCA_018902005.1), LOM (GCA_005234155.1), A1 (GCA_014217455.1), B17721 (GCA_016772175.1), B11243 (GCA_003014415.1), B11245 (GCA_008275145.1), B12342 (GCA_016772155.1), and IFRC2087 (GCA_016809505.1). Gene annotation was performed on the Companion server (http://companion.sanger.ac.uk, accessed on 9 March 2024). A maximum likelihood phylogenomic tree was constructed with OrthoFinder v4.0 software, utilizing a core-proteome-based phylogenomic analysis (CPBP) to obtain the clusters of orthologous groups of proteins (COGs) from the proteome of each organism [18]. 

The pan-genome analysis, encompassing the core genome, character genome, and accessory genome, was based on the annotated amino acid sequences of the following isolates: B8441, B11220, B11221, IFRC2087, B11243, B11245, B12342, and 20–1498. The pan-genome was constructed on the Orthovenn3 server [19], which incorporates the OrthoFinder algorithm. The phylogenomic tree was edited with Interactive Tree of Life v5 (iTOL) [20].

### 2.7. Phylogenetic and Comparative Analysis of the Erg11 and Fks1 Proteins

The phylogenetic trees were constructed for the Erg11 and Fks1 proteins based on their amino acid sequences. These were downloaded from the NCBI database and were comprised of different species of *Candida*, *Saccharomyces cerevisiae* S288C, and *Yarrowia lipolytica* CLIB122, which served as the outgroup species. The access numbers for Erg11 were the following: for *Candida auris*, 20–26 (000164700.1*), 20–32 (000136700.1*), B11220 (QEO20389.1), B11809 (000072800.1*), A1 (000324100.1*), LOM (000326000.1*), 20–1498 (000047500.1*), B11243 (PSK75255.1), B11245 (QEL61552.1), and IFRC2087 (QRG39199.1); for *Candida albicans*, SC5314 (XP_716761.1); for *Candida tropicalis*, MYA 3404 (XP_002550985.1); for *Candida parapsilosis*, ATCC 22019 (ACT67904.1); for *Candida lusitaniae*, ATCC 42720 (XP_002614916.1) and CBS 6936 (OVF10151.1); for *Candida haemulonii*, B11899 (XP_025344294.1) and LIP Ch2 (QOU12110.1); for *Candida duobushaemulonii*, B09383 (XP_025336625.1) and LIP Ch8 (QOU12108.1); for *Candida pseudohaemulonii*, B12108 (XP_024711630.1); for *Candida glabrata*, CBS 138 (XP_445876.1); for *Saccharomyces cerevisiae*, S288C (NP_011871.1); and for *Yarrowia lipolytica*, CLIB122 (XP_500518.1). The access numbers for Fks1 were the following: for *Candida auris*, 20–26 (000117700.1*), 20–32 (000098200.1*), B13916 (000299100.1*), B11220 (QEO20537.1), B11809 (000326000.1*), A1 (000218400.1*), LOM (000219600.1*), 20–1498 (000518900.1*), B11243 (PSK74959.1), and IFRC2087 (QRG37633.1); for *Candida albicans*, SC5314 (XP_721429.2); for *Candida tropicalis*, ATCC 750 (ACF22801.2); for *Candida orthopsilosis*, Co 90–125 (XP_003867907.1); for *Candida lusitaniae*, L17 (KAF5212065.1) and P5 (QFZ43597.1); for *Candida haemulonii*, B11899 (XP_025339819.1); for *Candida duobushaemulonii*, B09383 (XP_025335019); for *Candida pseudohaemulonii*, B12108 (XP_024714055.1); for *Candida glabrata*, M6 (KAI8387093.1); for *Saccharomyces cerevisiae*, S288C (NP_013446.1); and for *Yarrowia lipolytica*, CLIB122 (XP_504213.2). The alignment was generated by the CLUSTAL W v7 program available via MEGA7 software, and the phylogram was constructed with the maximum likelihood method and the Le and Gascuel + G model available via MEGA 7 software [21] by performing 1000 bootstrap replicates. The phylogenetic trees of the Erg11 and Fks1 proteins were edited with iTOL [20]. The antifungal profile and point mutations of the isolates used in the analysis and the phylogenetic trees of the *C. auris* Erg11 and Fks1 proteins are shown in Table S1 (Supplementary Material).

### 2.8. Modeling the Erg11 Protein from Candida auris 20–1498

The 3D structure of the *Candida auris* 20–1498 Erg11 protein was initially generated with the homology modeling technique, employing the Erg11 protein of *Candida albicans* with RCSB PDB ID: 5fsa as the template [22]. The analysis was conducted using Modeller v10.1 software [23], which is widely used to generate comparative models. Ten models were generated, and the best model was selected based on the lowest molpdf score. The results were validated with a Ramachandran plot, finding a greater percentage of residues located in favorable regions [24].

### 2.9. Molecular-Docking Study of Some Azoles on the Erg11 Protein

To explore the affinity of lanosterol, mevalonate, and some azoles for Erg11, docking simulations were carried out on the 3D structure of the *Candida auris* 20–1498 Erg11 protein. The lanosterol substrate of the Erg11 enzyme served as the positive control and mevalonate as the negative control. Subsequently, the affinity of some azoles (e.g., fluconazole and voriconazole) for Erg11 was examined. The docking simulations were performed using Autodock vina v4.2 [25].

## 3. Results

### 3.1. C. auris 20–1498 Genome Assembly and Annotation

The sequencing of the genome of *C. auris* 20–1498 showed a draft genome of 70 contigs, a total genome size of 12.86 Mbp, an N50 value of 1.6 Mbp, and an average guanine-cytosine (GC) content of 45.5% (Table 1).

### 3.2. Phylogenetic Tree and Pan-Genome of Candida auris

**Figure 1:**
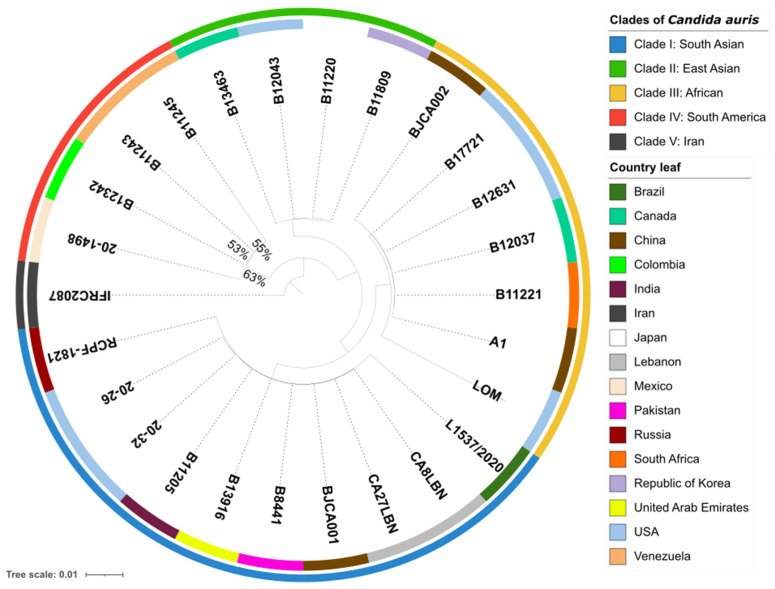
Phylogenomic tree of 26 strains of *Candida auris*, constructed using OrthoFinder with an orthologous gene model by performing 1000 bootstrap replicates. The 141,635 genes evaluated form a total of 5501 orthogroups, of which 4723 consist entirely of single-copy orthogroup genes existing in all the species. The outermost perimeter illustrates the geographic region corresponding to each of the five clades: clade I (blue), clade II (green), clade III (yellow), clade IV (red), and clade V (black). The labels of the leaves indicate the ID of each strain, and the color of each leaf refers to the country of origin of the strain.

To examine the phylogenetic relationship between *C. auris* 20–1498 and 25 strains of *C. auris* from different clades, single-copy orthologs in 26 sequenced genomes were identified by using OrthoFinder v2.5.4, which assigned 141,635 genes (99.8% of the total) to 5501 orthogroups. A total of 50% of all the genes were in orthogroups with 26 or more genes (G_50_ = 26) and were contained in the largest 2685 orthogroups (O_50_ = 2685). There were 5014 orthogroups among all the species, and 4723 of these consisted entirely of single-copy genes. Of the five known clades of *C. auris* in the world [4,5], the whole-genome sequencing of the isolate from Mexico confirmed that it was genetically closest to clade IV (containing strains endemic to South America) (Figure 1).

**Figure 2:**
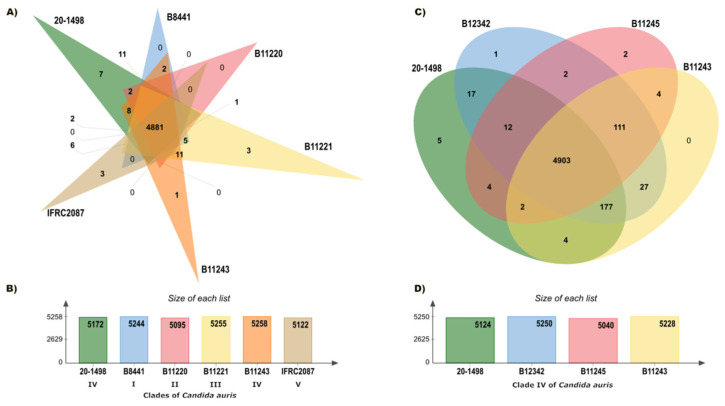
Summary of the pan-genome analysis of the orthologs carried out on the OrthoVenn3 server. (**A**) Venn diagram showing the distribution of orthologous clusters among the five clades of *C. auris*. (**B**) Summary of the proteins found in each strain of the five clades. (**C**) Venn diagram illustrating the distribution of orthologous clusters among the strains of *C. auris* in clade IV. (**D**) Summary of the proteins detected in each strain of clade IV.

The pan-genome analysis, conducted on the OrthoVenn3 server, created an orthologous clustering of the predicted proteins of the *C. auris* clades. *C. auris* IFRC2087 has a smaller proteome than the other isolates (*C. auris* B8441, *C. auris* B11220, *C. auris* B11221, *C. auris* B11243, and *C. auris* 20–1498). Based on 32,512 proteins, the OrthoVenn3 clustering displayed 5330 clusters, 4652 of which were single-copy clusters. Of the total number (in all 5 clades), those with at least 1 representative isolate of *C. auris* constituted 4881 core clusters (the core genome) (Figure 2A,B). A more limited comparison was performed between *C. auris* 20–1498 and three *C. auris* isolates from clade IV: *C. auris* B11243, *C. auris* B11245, and *C. auris* B12342. The evaluation of 21,925 proteins afforded 5271 clusters, of which 4749 were single-copy clusters and 4903 were core clusters (Figure 2C,D).

An evaluation was performed of the relation between the core genome of *C. auris* 20–1498 and two different groups: (1) all of the strains of the five clades, and (2) three strains in clade IV. At least 22 more clusters were observed when comparing the core genome of *C. auris* 20–1498 with the second versus the first group. The strain most closely linked to *C. auris* 20–1498 was *C. auris* B12342 from clade IV. According to the similarity matrix, the genomes of these two strains formed a great number of clusters (5222). The pairwise heatmaps of the number of overlapping clusters between each pair of *C. auris* species from the five different clades (Figure S1) and of the number of overlapping clusters between each pair of *C. auris* species from clade IV (Figure S2) are included in the Supplementary Material.

### 3.3. Phylogenetic and Comparative Analysis of Erg11 and Fks1 Proteins

**Figure 3:**
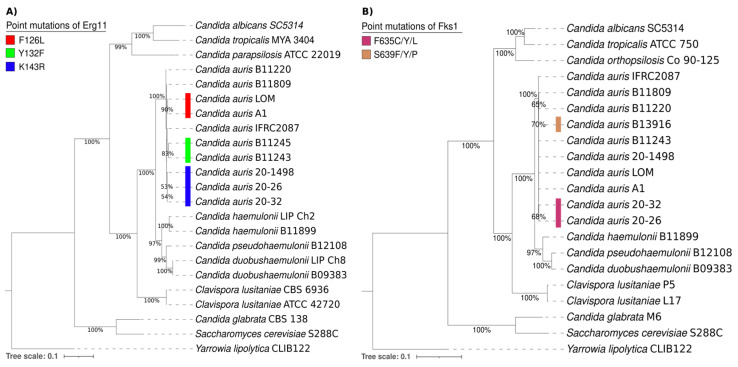
Phylogenetic trees of the Erg11 (**A**) and Fks1 proteins (**B**) and their putative orthologs in various *Candida* spp., 1 *Saccharomyces cerevisiae S288C* strain, and 1 *Yarrowia lipolytica CLIB122* strain as an outgroup. The point mutations of Erg11 and Fks1 from *C. auris* are illustrated by distinct colors on the phylogenetic trees. For the Erg11 point mutations, F126L is depicted in red, Y132F in green, and K143R in blue. For the Fks1 point mutations, F635C/Y/L is portrayed in purple and S639Y/P/F in brown.

The phylogenetic tree of the Erg11 proteins from different yeasts consisted of 21 amino acid sequences of *Candida* spp., 1 of *Saccharomyces cerevisiae* S288C, and 1 of *Yarrowia lipolytica* CLIB122 as an outgroup. It was generated with the maximum likelihood method and the Le and Gascuel + G model (parameter = 0.9595) using MEGA7 software by performing 1000 bootstrap replicates (Figure 3A). On the other hand, the phylogenetic tree of the Fks1 proteins from different yeasts was comprised of 19 amino acid sequences of *Candida* spp., 1 of *Saccharomyces cerevisiae* S288C, and 1 of *Yarrowia lipolytica* CLIB122 as an outgroup. It was also generated with the maximum likelihood method and the Le and Gascuel + G model (parameter = 0.7018) using MEGA7 software by performing 1000 bootstrap replicates (Figure 3B). 

Overall, the comparison of the amino acid sequences of the Erg11 and Fks1 proteins shows a close relationship between *C. auris*, *C. haemulonii*, *C. duobushaemulonii*, and *C. pseudohaemulonii* and justifies their grouping into a single clade. The present phylogenetic analysis confirmed that the Erg11 and Fks1 proteins from *C. haemulonii*, *C. duobushaemulonii*, and *C. pseudohaemulonii* are closely related to the same proteins in *C. auris* [26]. Moreover, the genotypes were determined for specific mutations in the Erg11 proteins (Y132F, K143R, and F126L) of *C. auris* strains associated with resistance to azoles. Based on the results, the K143R mutation detected in the lanosterol 14-alpha-demethylase (Erg11) of the *C. auris* 20–1498 isolate is probably related to the clinically observed resistance of this strain to fluconazole [10]. The K143R mutation has been predominately identified in clade I and in a few isolates from clade IV [1,3,27,28].

Regarding the S639Y/P/F and F635C/Y/L mutations in 1,3-beta-D-glucan synthase (Fks1), the most frequent mutation is S639P in isolates from clade IV. Likewise, S639F and S639Y are the most commonly identified mutations in micafungin-resistant isolates from clades I and III [3,27]. There are also reports of a F635C/Y/L mutation in isolates from clade I [28,29]. The fact that none of these mutations occurred in the *C. auris* 20–1498 strain is in accordance with the clinically observed susceptibility of the yeast to caspofungin [10]. 

### 3.4. Modeling the Candida auris 20–1498 Erg11 Protein, and Its Use for the Molecular Docking of Some Azoles

**Figure 4:**
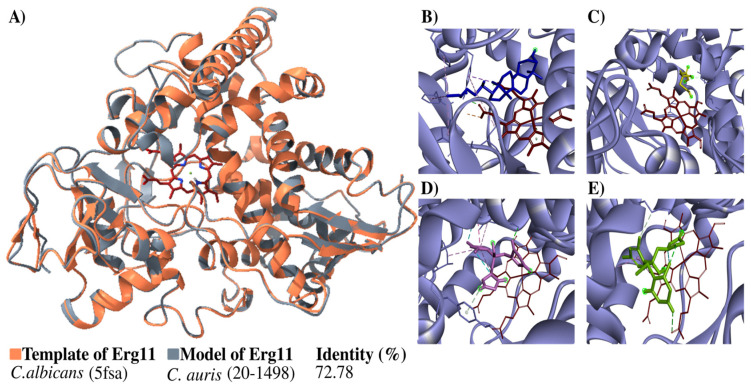
Modeling the *C. auris* 20–1498 Erg11 protein and its use for the molecular docking of some azoles. (**A**) The schematic illustration portrays the binding mode of the ligand with the Erg11 protein. The flat ribbon representation of the Erg11 of *Candida* spp. reveals the overlapping of *C. albicans* Erg11 (PDB: 5fsa) (orange) with *C. auris* 20–1498 Erg11 (gray). The heme as the prosthetic group is depicted in red (stick representation). The percentage of identity with their respective template is listed for the model. The predicted binding mode on *C. auris* 20–1498 Erg11 is shown for lanosterol (blue) (**B**), mevalonate (yellow) (**C**), fluconazole (pink) (**D**), and voriconazole (green) (**E**).

The structure of the Erg11 protein was built in 3D and then verified (Figure 4). The structure generated overlap with the template, indicating a high percentage of identity (72.78%) with it (Figure 4A). The Ramachandran plots constructed for the *Candida auris* 20–1498 Erg11 protein showed 89.6% of the residues located in favorable regions, demonstrating the reliability of the structure.

The next step was a coupling analysis to test the hypothesis that the protein had a higher affinity for the natural substrate lanosterol than for mevalonate (the negative control) and antifungals belonging to the azole family (Figure 4B–E). The docking study of the binding mode between lanosterol and the Erg11 protein (Table 2) evidenced a low binding energy value (high affinity). Meanwhile, there were higher binding energy values (lower affinity) for fluconazole and voriconazole on the Erg11 protein. The binding energies between the different ligands and the Erg11 protein K143 (wild-type) and R143 (substitution mutation) were also determined with a coupling analysis (Table 2) (Figure S3).

The models of the wild-type (K143) and mutant (K143R) Erg11 protein of *C. auris* 20–1498 are illustrated in Figure S3 (Supplementary Material). The docking results of the binding mode of lanosterol, mevalonate, fluconazole, and voriconazole at the catalytic site of each of these Erg11 proteins are shown in Table S2 (Supplementary Material).

### 3.5. Phenotypic Characteristics: Thermotolerance and Halotolerance

**Figure 5:**
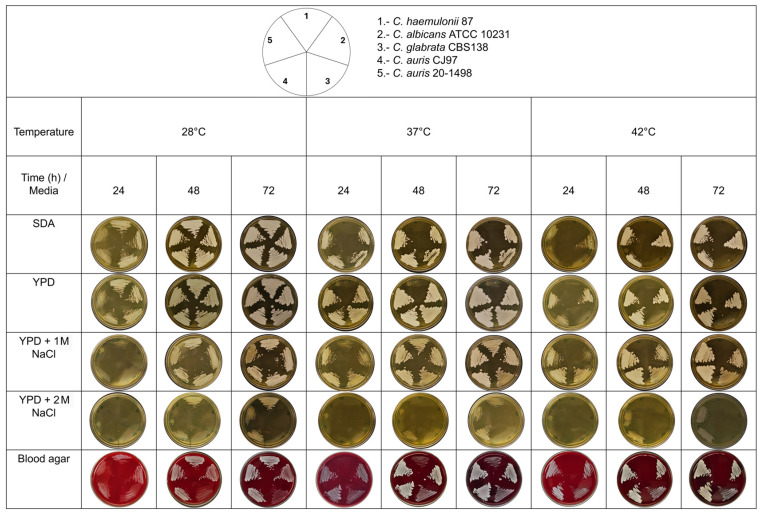
Thermotolerance and halotolerance phenotype of the Mexican *C. auris* 20–1498 compared to *C. auris* CJ97, *C. albicans* ATCC 10231, *C. glabrata* CBS 138, and *C. haemulonii* 87. The yeasts were grown in YPD broth under constant shaking at 28 °C until reaching the early stationary phase of growth (~15 h). The inoculum was adjusted to As_600_ = 0.5 with sterile YPD medium, and 5 μL of each strain was inoculated onto the corresponding culture media and streaked with a microbiological loop. The cultures were incubated at different temperatures (28, 37, and 42 °C), and the yeast growth was recorded every 24 h for 3 days. SDA, YPD, YPD-1 M NaCl, YPD-2 M NaCl, and blood agar served as the solid culture media [11].

Thermotolerance and halotolerance have been described as characteristics that could help *C. auris* to survive in hospital environments. These characteristics may also help differentiate strains from the Metschnikowiaceae family, specifically between the *C. auris* clade and the *C. haemulonii* complex [11]. *C. auris* 20–1498 was found to be thermoresistant when incubated at 42 °C, unlike *C. haemulonii* 87, which was thermosensitive when incubated in poor media (YPD and SDA) at 37 °C and tolerant in rich media (e.g., BHI agar and blood agar) at 37 °C (Figure 5). Unlike *C. albicans* and *C. glabrata*, *C. auris* 20–1498 showed halotolerance, as it could resist NaCl at concentrations of 1 and 2 M.

## 4. Discussion

*C. auris*, an emerging fungal pathogen around the world, has been a challenge for major hospitals around the world because of its resistance to multiple antifungal agents. As a consequence, the treatment options are severely limited [30]. 

Before the first isolate of *C. auris* was detected in Mexico in 2020 [10], the strain was absent from national epidemiological and etiological reports on candidiasis [31]. The current contribution is the first attempt to define the molecular features of the *C. auris* isolate and carry out a phylogenomic search for mutations linked to its antifungal resistance. The short-read sequencing technique was utilized to produce a complete genome sequence of this pathogenic strain. 

Whole-genome sequencing demonstrated the close relationship between the *C. auris* 20–1498 isolate and three strains of *C. auris* herein used to represent clade IV (containing strains endemic to South America). *C. auris* 20–1498 is more closely related to *C. auris* B12342 from Colombia than to *C. auris* B11243 and B11245 from Venezuela. The data here generated by whole-genome sequencing will serve to explore the population structure of *C. auris* 20–1498 and gain further insights into why certain strains are responsible for the multidrug resistance of a given clade. Such information should facilitate monitoring of the global dissemination of drug-resistant strains [32].

The pan-genomic analysis of *C. auris* strains in clade IV revealed an exclusive genome of the *C. auris* 20–1490 strain with five clusters. Each cluster contains different genes that encode proteins distinct from those encoded by other clusters. Thus, each cluster likely has a distinct function, defined by the assigned Gene Ontology (GO) term and Swiss-Prot Hit. Cluster 1 contains two proteins (with accession numbers 000009700.1 and 000309600.1) assigned the term GO:0005351 (F:carbohydrate:proton symporter activity) and the Swiss-Prot Hit code A0A1D8PCL1 (high-affinity glucose transporter). Cluster 2 also contains two proteins (with accession numbers 000076100.1 and 000194600.1) given the term GO:0055085 (P:transmembrane transport; IDA:SGD) and the Swiss-Prot Hit code P13587 (sodium transport ATPase 1). Cluster 3 contains two proteins (with accession numbers 000353300.1 and 000370500.1) designated by the term GO:0005524 (F: ATP binding) and the Swiss-Prot Hit code: P53623 (heat shock protein 70). Finally, clusters 4 and 5 each had two hypothetical proteins: the former 000305000.1 and 000326000.1, and the latter 000119800.1 and 000521300.1. These were not furnished a GO term or Swiss-Prot Hit code.

Because mutations vary in relation to the clade and country of origin of a species, mutations are examined in relation to the antifungal resistance of each specific clade. Based on the minimum inhibitory concentration (MIC) breakpoints recommended by the CDC and the Clinical Laboratory Standard Institute (CLSI, supplement M60) [33], *C. auris* 20–1498 is resistant to fluconazole (≥64 µg/mL) and amphotericin B (≥2 µg/mL) but susceptible to caspofungin (≥0.5 µg/mL) [10]. When the phenotypic test was matched with the genotypic results of the *C. auris* 20–1498 isolate, it was not surprising to find the K143R mutation, which has been reported in a few fluconazole-resistant isolates of *C. auris* belonging to clade IV. This substitution is linked to the elevated MICs of azoles [3,34]. According to the docking study, the Erg11 protein has greater affinity for its substrate (lanosterol) than for two of the main antifungals belonging to the azole group (fluconazole and voriconazole).

Two other mutations in Fsk1 (S639F and F635) are linked to multidrug resistance in various *C. auris* strains. Neither one was found in *C. auris* 20–1498. S639F has been detected in the Fks1 of the multidrug-resistant *C. auris* B13916 strain, while F635C has been identified in the pandrug-resistant *C. auris* 20–26 and *C. auris* 20–32 strains. For the latter strains, the MIC of echinocandin is high [28]. The docking simulations with the Fks1 protein are in agreement with the results concerning the caspofungin sensitivity. The Mexican patient evolved favorably with systemic antifungal therapy with caspofungin [10].

Studies on evolved strains of *C. auris* have revealed multiple novel mechanisms of multidrug resistance [35]. With mutations in *ERG3* and *CIS2*, there is a mutation in the transcription factor TAC1b and an overexpression of the drug efflux pump Cdr1, leading to a higher MIC for echinocandin. Some of the mechanisms of resistance to amphotericin B are known to be related to the expression levels of genes in the ergosterol biosynthesis pathway. Evaluation of the reverse transcription PCR results demonstrated that Upc2 regulates *ERG11* expression and also activates the Mrr1/Mdr1 pathway [36]. In the current contribution, the point mutation V704L in Cdr1 was not detected in the genome of *C. auris* 20–1498. Future research can take advantage of the *C. auris* 20–1498 genome database herein generated in order to carry out gene expression studies with the aim of exploring new resistance mechanisms in this strain and other clade IV isolates.

The present analysis of the resistance mechanisms of *C. auris* focused on mutations in the *ERG11* and *FKS1* genes. Besides being the genes associated with resistance to azoles and echinocandins, which together with amphotericin B constitute the drugs of choice for antifungal treatment in Mexico, they are the markers present in the greatest abundance in the current gene databases and have been reported in practically all the clades of *C. auris* (I-V). Hence, research on these markers would allow for epidemiological and comparative analysis to be carried out, even without having sequenced the complete genome. 

In Mexico, 20–1498 is the first known strain of *C. auris* isolated from a patient, and no further information exists in this country on mutations in the Erg11 and Fks1 proteins in *C. auris*. It is very important to evaluate the latter proteins in relation to the antifungals recommended for the treatment of invasive candidiasis in Mexico, with fluconazole being the first drug of choice, followed by echinocandins, voriconazole, and amphotericin B [10]. The resulting information on the point mutations is important for the establishment of accurate antifungal resistance and antifungal susceptibility testing in healthcare settings. Such testing would be invaluable in determining appropriate therapeutic strategies. On the other hand, since the genome sequence of the *C. auris* 20–1498 strain has been deposited in the NCBI database, it can provide a model for further research on resistance, virulence factors, molecular epidemiology, therapeutic targets, and antifungal design.

According to the thermotolerance and halotolerance capacity found for the *C. auris* 20–1498 strain, it likely emerged from a natural reservoir, a conclusion supported by genomic evidence and the ecology of related fungal species [37]. Since pathogenic *C. auris* can tolerate high concentrations of salt, it likely evolved in niches of marine ecosystems [37]. Its other potential environmental sources include terrestrial and freshwater reservoirs, with specific niches in soil, plants, and animals. 

The characteristics of *C. auris* in relation to thermotolerance (at 40–42 °C) and halotolerance to NaCl have been analyzed [37,38]. It is suggested that thermotolerance and halotolerance would be advantageous for the survival of fungi on the skin, axilla, and groin, the most common sites of *C. auris* isolation in intra-hospital environments [37]. *C. auris* 20–1498 was isolated from an in-hospital environment, a hospital that months later was converted into a COVID-19 unit [39]. Therefore, this hospital should be monitored for the persistence of the same strain.

The current data can be used to study fungal biology and virulence in order to provide greater insight into the phylogenetic relationships between multidrug-resistant *C. auris* strains and to determine which genomic regions are associated with specific phenotypes. The information on the genome of *C. auris* will allow for further related research, such as comparative analysis and the evolution of the genomes, on the first *C. auris* strain isolated in Mexico and the one isolated months later in the same hospital in a patient with COVID-19 [37,38]. The focus on whole-genome sequencing and the identification of mutations linked to the drug resistance of fungi could lead to the discovery of new therapeutic targets as well as new antifungals capable of responding to the serious problem of the multidrug resistance of *C. auris*.

## 5. Conclusions

The first isolate of *C. auris* detected in Mexico is the 20–1498 strain. It has a total genome size of 12.86 Mbp and an average guanine-cytosine (GC) content of 45.5%. Genome annotation revealed a total of 5432 genes encoding 5515 proteins. The genomic analysis demonstrated that the *C. auris* 20–1498 strain belongs to clade IV (containing strains endemic to South America). Of the two genes (*ERG11* and *FKS1*) associated with drug resistance in *C. auris*, a mutation was found in the K143R gene located in a mutation hotspot of *ERG11* (lanosterol 14-α-demethylase), an antifungal drug target of azoles. The Cdr1 point mutation V704L was not detected in the genome of *C. auris* 20–1498. The current results can be used to study the fungal biology and virulence in order to provide greater insight into the phylogenetic relationships between multidrug-resistant *C. auris* strains and to determine which genomic regions are associated with specific phenotypes. The focus on whole-genome sequencing and the identification of mutations linked to the drug resistance of fungi could lead to the discovery of new therapeutic targets and new antifungal compounds.

## Figures and Tables

**Table 1:** Summary of the *C. auris* 20–1498 annotated genome assembly.

Features	Values
Quality of reads	QC ^1^ > 30
Genome assembly size (bp)	12,869,713
Number of contigs	70
N50 (bp)	1,604,526
Genome coverage	99.68
GC content (%)	45.5
Number of predicted genes	5432
Number of coding genes	5304
Number of genes with multiple CDSs	565
Number of hypothetical proteins	5515

^1^ Quality control. GC, guanine-cytosine; CDS, coding sequences.

**Table 2:** Docking results of the binding mode between lanosterol, mevalonate, fluconazole, and voriconazole at the catalytic site of the *C. auris* 20–1498 Erg11 protein.

Molecules	Binding Energy (kcal/mol)	Interacting Residues	Interactions
Lanosterol	K143; R143 WT; M −12; −10.4	Val A: 304, HEM A: 525, Gly A: 307, Leu A: 376, Met A: 504, Pro A: 230, Leu A: 121, Phe A: 380, His A: 377, Phe A: 233, Ser A: 378, Tyr A: 118, Thr A: 122, Ile A: 131, Gly A: 303, Phe A: 126, Leu A: 300	Van Der Waals Pi-sigma Alkyl Pi-alkyl
Mevalonate	−5–5; −4.4	Gly A: 308, Phe A: 126, Tyr A: 132, Ile A: 131, Val A: 304, HEM A: 525, Leu A: 300, Gly A: 303, Gly A: 307	Van Der Waals Carbon hydrogen bond
Fluconazole	−8.8; −8.6	Leu A: 300, Val A: 304, Gly A: 303, Ile A: 131, Tyr A: 132, HEM A: 525, Phe A: 228, Tyr A: 118, Leu A: 376, Pro A: 375, Thr A: 311, His A: 310, Gly A: 307, Phe A: 126	Van Der Waals Conventional hydrogen bond Halogen (fluorine) Pi-donor hydrogen bond Amide-pi stacked Pi-alkyl
Voriconazole	−9.5; −7.3	Leu A: 376, Ile A: 379, Arg A: 381, Thr A: 311, Gly A: 308, Ile A: 131, Leu A: 300, Val A: 304, Gly A: 303, Phe A: 126, Thr A:122, Gly A: 307, Tyr A: 132, HEM A: 525	Van Der Waals Conventional hydrogen bond Carbon hydrogen bond Alkyl Pi-alkyl

The binding energy is expressed as kCal/mol (ΔG). Lanosterol, the natural substrate of the Erg11 enzyme, served as the positive binding control and mevalonate as the negative binding control. WT, wild-type; M, substitution mutation.

## Data Availability

The *Candida auris* 20–1498 genome sequence herein generated was deposited in the GenBank BioProject (PRJNA1013603) with genome annotation and BioSample ID (SAMN39051800).

## Supplementary Materials

The following supporting information can be downloaded at: https://www.mdpi.com/article/10.3390/jof10060392/s1.
