# Supplementary material for: Comparative Genomics of the First Resistant Candida auris Strain Isolated in Mexico: Phylogenomic and Pan-Genomic Analysis and Mutations Associated with Antifungal Resistance
Source: J Fungi (Basel). 2024 May 30;10(6):392. doi: 10.3390/jof10060392 (PMC11204476; doi:10.3390/jof10060392)
Supplement: Supplementary file 1 [file jof-10-00392-s001.zip › jof-3010865-supplementary.pdf]

Supplementary Material
Journal of Fungi
"Comparative genomics of the first resistant *Candida auris* strain isolated in Mexico: phylogenomic and pan-genomic analysis, and mutations associated with antifungal resistance", by
Arturo Casimiro-Ramos, Celia Bautista Crescencio, Alvaro Vidal Montiel, Gloria M. González, J. Alfredo Hernández-García, César Hernández-Rodríguez, and Lourdes Villa-Tanaca, which we are resubmitting to your prestig

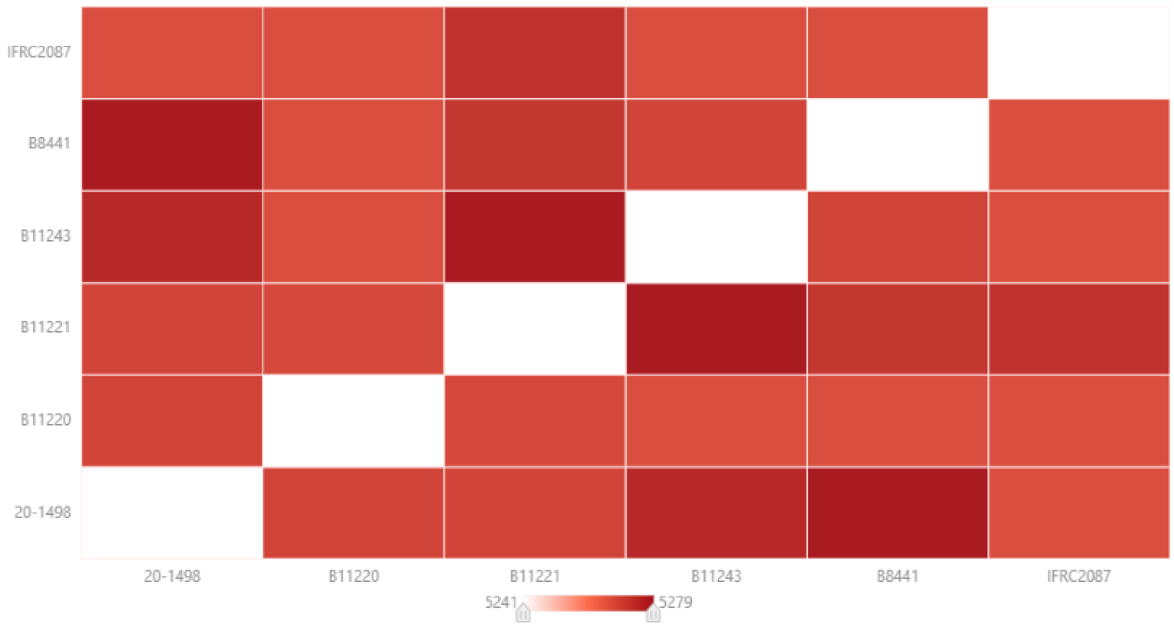

Figure S1. Pairwise heatmap of the number of overlapping clusters between each pair of species from five different *Candia auris* clades.

Finally, based on the similarity matrix of the *C. auris* species in clade IV, the heatmap module showed the shared orthologous gene clusters between each pair of species. It turns out that *C. auris* 20-1498 and *C. auris* B12342 had greater similarity and formed a higher number of clusters (5,222 clusters, Figure 2) than *C. auris* 20-1498 with either of the other three *C. auris* strains belonging to clade IV.

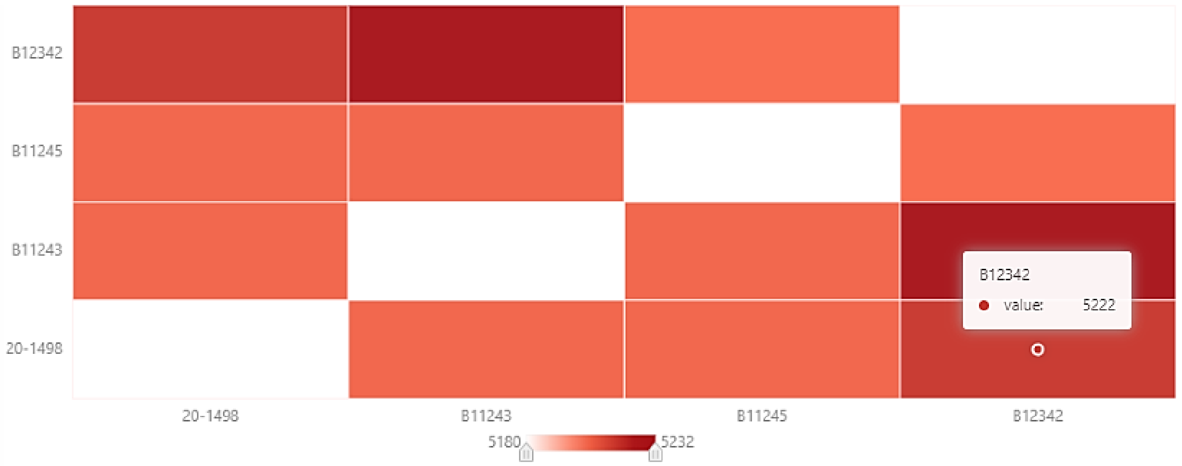

Figure S2. Pairwise heatmap of the number of overlapping clusters between each pair of species from the *Candia auris* species in clade IV.

Table S1. Antifungal profile and point mutations of the isolates used in the analysis and phylogenetic trees of the *C. auris* Erg11 and Fks1 proteins.

Clade		Antifungal profiles				Point mutation Erg11	Point mutation Fks1
		FCZ	AMB	MCF	CSF		
Clade I	20-26	R	S	R	R	K143R	F635C
	20-32	R	R	R	R	K143R	F635C
	B13916	R	R	R	-	-	S639F
Clade II	B11220	S	S	S	-	-	-
	B11809	R	S	S	-	-	-
Clade III	LOM	R	S	S	-	F126L	-
	A1	*	*	*	*	F126L	-
Clade IV	20-1498	R	R	-	S	K143R	-
	B11243	R	S	S	S	Y132F	-
	B11245	R	S	S	S	Y132F	-
Clade V	IFRC2087	S	S	S	-	-	-

Fluconazole (FCZ), amphotericin B (AMB), micafungin (MCF), caspofungin (CSF), resistant to antifungal (R), and susceptible to antifungal (S). * No data found about antifungal profiles.

At this time, there are no clinical MIC breakpoints reported for *C. auris*. However, the CDC has recommended breakpoints set at ≥32 µg/mL for fluconazole, ≥2 µg/mL for amphotericin B, ≥2 µg/mL for caspofungin, and ≥4 µg/mL for micafungin.

References are listed based on information for each *Candida auris* strain used in the study.

C. auris 20-26<br>*C. auris* 20-32	Jacobs, S. E., Jacobs, J. L., Dennis, E. K., Taimur, S., Rana, M., Patel, D., Gitman, M., Patel, G., Schaefer, S., Iyer, K., Moon, J., Adams, V., Lerner, P., Walsh, T. J., Zhu, Y., Anower, M. R., Vaidya, M. M., Chaturvedi, S., & Chaturvedi, V. (2022). *Candida auris* Pan-Drug-Resistant to Four Classes of Antifungal Agents. Antimicrobial Agents and Chemotherapy, 66(7), e0005322. https://doi.org/10.1128/aac.00053-22
C. auris B13916<br>*C. auris* B11220<br>*C. auris* B11809	Chow, N. A., Muñoz, J. F., Gade, L., Berkow, E. L., Li, X., Welsh, R. M., Forsberg, K., Lockhart, S. R., Adam, R., Alanio, A., Alastruey-Izquierdo, A., Althawadi, S., Araúz, A. B., Ben-Ami, R., Bharat, A., Calvo, B., Desnos-Ollivier, M., Escandón, P., Gardam, D., Gunturu, R., … Cuomo, C. A. (2020). Tracing the Evolutionary History and Global Expansion of *Candida auris* Using Population Genomic Analyses. mBio, 11(2), e03364-19. https://doi.org/10.1128/mBio.03364-19
C. auris B11220	Satoh, K., Makimura, K., Hasumi, Y., Nishiyama, Y., Uchida, K., & Yamaguchi, H. (2009). *Candida auris* sp. nov., a novel ascomycetous yeast isolated from the external ear canal of an inpatient in a Japanese hospital. Microbiology and Immunology, 53(1), 41–44. https://doi.org/10.1111/j.1348-0421.2008.00083.x
C. auris LOM<br>*C. auris* A1	Long, S. W., Olsen, R. J., Nguyen, H. A. T., Ojeda Saavedra, M., & Musser, J. M. (2019). Draft Genome Sequence of *Candida auris* Strain LOM, a Human Clinical Isolate from Greater Metropolitan Houston, Texas. Microbiology Resource announcements, 8(25), e00532-19. https://doi.org/10.1128/MRA.00532-19<br><br>Long, S. W., Ojeda Saavedra, M., Christensen, P. A., Musser, J. M., & Olsen, R. J. (2020). Human Infections Caused by Clonally Related African Clade (Clade III) Strains of *Candida auris* in the Greater Houston Region. Journal of Clinical Microbiology, 58(7), e02063-19. https://doi.org/10.1128/JCM.02063-19<br><br>de Jong, A. W., Francisco, E. C., de Almeida, J. N., Jr, Brandão, I. B., Pereira, F. M., Dias, P. H. P., de Miranda Costa, M. M., de Souza Jordão, R. T., Vu, D., Colombo, A. L., & Hagen, F. (2021). Nanopore Genome Sequencing and Variant Analysis of the Susceptible *Candida auris* Strain L1537/2020, Salvador, Brazil. Mycopathologia, 186(6), 883–887. https://doi.org/10.1007/s11046-021-00593-7
C. auris IFRC8026	Chow, N. A., de Groot, T., Badali, H., Abastabar, M., Chiller, T. M., & Meis, J. F. (2019). Potential Fifth Clade of *Candida auris*, Iran, 2018. Emerging infectious diseases, 25(9), 1780–1781. https://doi.org/10.3201/eid2509.190686

	Abastabar, M., Haghani, I., Ahangarkani, F., Rezai, M. S., Taghizadeh Armaki, M., Roodgari, S., Kiakojuri, K., Al-Hatmi, A. M. S., Meis, J. F., & Badali, H. (2019). *Candida auris* otomycosis in Iran and review of recent literature. Mycoses, 62(2), 101–105. https://doi.org/10.1111/myc.12886
C. auris 20-1498	Ayala-Gaytán, J. J., Montoya, A. M., Martínez-Resendez, M. F., Guajardo-Lara, C. E., de J Treviño-Rangel, R., Salazar-Cavazos, L., Llaca-Díaz, J. M., & González, G. M. (2021). First case of *Candida auris* isolated from the bloodstream of a Mexican patient with serious gastrointestinal complications from severe endometriosis. Infection, 49(3), 523–525. https://doi.org/10.1007/s15010-020-01525-1
C. auris B11243<br>*C. auris* B11245	Lockhart, S. R., Etienne, K. A., Vallabhaneni, S., Farooqi, J., Chowdhary, A., Govender, N. P., Colombo, A. L., Calvo, B., Cuomo, C. A., Desjardins, C. A., Berkow, E. L., Castanheira, M., Magobo, R. E., Jabeen, K., Asghar, R. J., Meis, J. F., Jackson, B., Chiller, T., & Litvintseva, A. P. (2017). Simultaneous Emergence of Multidrug-Resistant *Candida auris* on 3 Continents Confirmed by Whole-Genome Sequencing and Epidemiological Analyses. Clinical infectious diseases: an official publication of the Infectious Diseases Society of America, 64(2), 134–140. https://doi.org/10.1093/cid/ciw691

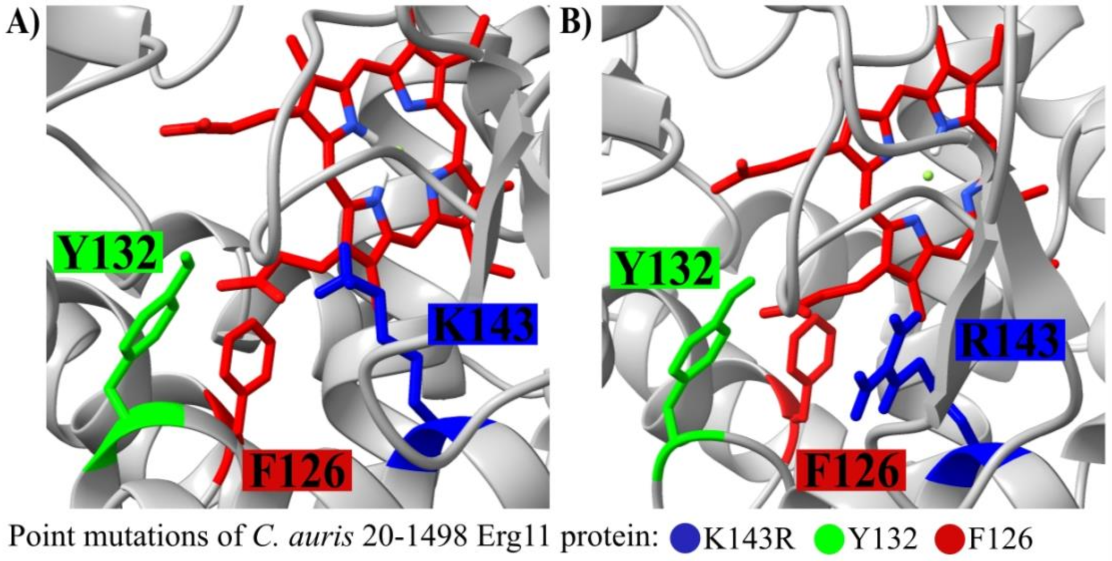

Figure S3. Models of *C. auris* 20-1498 Erg11 proteins. **A)** Model of Erg11 protein (K143 wild-type) and **B)** Model of Erg11 protein (with K143R mutation).

Table S2. Docking results of the binding mode of lanosterol, mevalonate, fluconazole, and voriconazole at the catalytic site of the *C. auris* 20-1498 Erg11 protein and wild-type Erg11 protein.

**Erg11 protein**	***C. auris* (K143)**	***C. auris* 20-1498 (R143)**
**Molecules**	**Binding energy (kcal/mol)**	
Lanosterol	-12	-10.4
Mevalonate	-5.5	-4.4
Fluconazole	-8.8	-8.6
Voriconazole	-9.5	-7.3

The docking results of the binding mode (Table S2) for Erg11 (K143) and Erg11 (R143) showed a similar binding energy pattern for lanosterol, mevalonate, and fluconazole. There was a lower binding energy value (reflecting higher affinity) between lanosterol and the Erg11 proteins.

Interestingly, a distinct binding energy pattern was observed for voriconazole, which showed a lower binding energy value (higher affinity) for the Erg11 (K143) protein than the Erg11 (R143) protein.
